# Family-Centred Early Hearing Detection and Intervention in the African Context: Relevance and Responsiveness to African Culture

**DOI:** 10.3390/audiolres15020030

**Published:** 2025-03-15

**Authors:** Katijah Khoza-Shangase

**Affiliations:** Audiology Department, Faculty of Humanities, School of Human & Community Development, University of the Witwatersrand, Johannesburg 2050, South Africa; Katijah.Khoza-Shangase@wits.ac.za

**Keywords:** family-centred care, early hearing detection, intervention, Africa, cultural responsiveness, tele-audiology, audiology, community-based care

## Abstract

Family-centred early hearing detection and intervention (FC-EHDI) is an established framework globally recognized for its emphasis on family involvement in supporting children who are deaf and hard of hearing (DHH). In the African context, unique sociocultural and systemic challenges necessitate tailored approaches to ensure effective implementation. This narrative review explores the relevance of FC-EHDI in Africa, highlights barriers to its implementation, and offers recommendations for creating sustainable and culturally aligned interventions. A narrative review methodology synthesizing evidence from African countries to examine the intersection of FC-EHDI with cultural practices, systemic barriers, and opportunities for innovation was adopted. Databases including PubMed, Scopus, Web of Science, and Google Scholar were searched for peer-reviewed journal articles, books, and reports published between 2000 and 2024. Keywords included “family-centred care”, “EHDI”, “Africa”, “cultural responsiveness”, and “early hearing detection and intervention”. Studies were included if they addressed EHDI in African contexts, explored family-centred approaches, or provided barriers and recommendations specific to the region. Thematic analysis was employed to synthesize findings into barriers, evidence, and strategies for FC-EHDI implementation. Data were extracted and analysed thematically to identify patterns and gaps in knowledge. Key challenges identified include resource limitations, economic constraints, linguistic and cultural diversity, and fragmented healthcare systems. Evidence highlights the effectiveness of community-based care, linguistic inclusivity, and culturally tailored interventions in enhancing family engagement and programme outcomes. Recommendations focus on leveraging technology, interdisciplinary collaboration, and policy advocacy. FC-EHDI offers a transformative approach to addressing DHH African children by integrating family involvement, cultural responsiveness, and systemic innovations. Future efforts should emphasize technology, scalable models, and family empowerment to create sustainable and equitable services.

## 1. Introduction

Early hearing detection and intervention (EHDI) is critical in identifying DHH children and initiating timely interventions to optimize communication, cognitive, and social development [1]. Hearing impairment, if left undetected, can lead to significant delays in speech and language acquisition, academic underachievement, and social isolation. These developmental setbacks not only affect the individual but also place a significant burden on families and healthcare systems [2,3,4,5,6]. The inclusion of families in the intervention process is recognized as a best practice globally, leading to the evolution of family-centred EHDI (FC-EHDI) [7,8,9]. This approach views the family as the primary decision-maker and partner in the intervention process, ensuring that interventions align with family values, culture, and needs. The evolving landscape of family-centred early intervention is reflected in the recently expanded Family-Centred Early Intervention for Deaf and Hard of Hearing (FCEI-DHH) Principles. These updated principles build on the foundation established by the 2013 Consensus Statement, reinforcing core values such as respect for diversity, informed decision-making by families, and collaborative partnerships between caregivers and professionals. A key advancement in the updated principles is their emphasis on cultural and global adaptability, ensuring that interventions are tailored to diverse contexts and that equitable access to services is prioritized. By incorporating these principles, early intervention frameworks can be strengthened to promote both family well-being and optimal developmental outcomes for children who are DHH. Given the cultural and systemic complexities of EHDI in Africa, aligning with these updated guidelines offers a valuable framework for enhancing service delivery and family support structures.

Family-centred care emphasizes the empowerment of caregivers, fostering their active involvement in decision-making, and acknowledging their unique insights into their child’s needs [10,11]. This approach aligns well with the principles of inclusive and holistic healthcare, where the well-being of the child is understood in the context of the family’s social, emotional, and cultural environment [12]. FC-EHDI is not merely an enhancement of clinical care but a paradigm shift that places families at the core of intervention strategies [13,14].

In Africa, where healthcare systems face challenges such as resource constraints, inequities in access, and a high burden of disease, implementation EHDI programmes has been limited [2,5,6,14,15]. These challenges are compounded by linguistic and cultural diversity, making universal models less effective [5,16]. Sub-Saharan Africa, in particular, has some of the highest rates of untreated hearing impairment globally, exacerbated by systemic issues such as a lack of trained audiologists, inadequate diagnostic infrastructure, and economic barriers [2,15,17,18]. In this context, FC-EHDI offers a promising solution by integrating cultural responsiveness and prioritizing family engagement, aligning well with African societal norms and values [13].

African societies are characterized by a collectivist culture where extended family and community play a significant role in caregiving [8,9,19,20,21]. This communal approach to child-rearing provides a strong foundation for implementing FC-EHDI [5]. By leveraging these cultural strengths, FC-EHDI can enhance the effectiveness of interventions and ensure they are sustainable within local contexts. Additionally, the linguistic diversity across the continent necessitates an approach that respects and incorporates the home languages of families, thereby improving communication and trust between caregivers and healthcare providers [22].

As healthcare systems in Africa strive to meet the United Nation’s Sustainable Development Goals (SDGs), particularly those related to health and well-being, addressing the needs of DHH children becomes a crucial component of equitable healthcare delivery. Established in 2015 by the United Nations, the SDGs are a set of 17 global objectives aimed at achieving a more sustainable and inclusive future by 2030. Among these, Goal 3—‘Good Health and Well-Being’—is especially relevant to audiology, as it calls for universal health coverage, access to quality essential healthcare services, and reductions in preventable mortality. Additionally, Goal 4—‘Quality Education’—emphasizes inclusive education, which directly impacts children with hearing loss. Ensuring early hearing detection and intervention aligns with these global targets by facilitating access to essential health services, promoting equity in education, and improving overall developmental outcomes. Given the resource constraints and health disparities in many African nations, embedding EHDI services within broader efforts to achieve the SDGs presents an opportunity to strengthen health systems and improve long-term outcomes for children who are deaf or hard of hearing. FC-EHDI has the potential to bridge the gaps in service delivery by addressing both the technical and cultural aspects of care, ensuring that interventions are not only accessible but also meaningful to the families they aim to serve.

## 2. Materials and Methods

This study employed a systematic approach to conducting a narrative review, ensuring structured collation and thematic synthesis of the existing literature. The process involved a systematic search and selection of relevant studies, followed by a narrative synthesis that categorized findings into themes related to barriers, supporting evidence, and recommendations for FC-EHDI in Africa. Rather than presenting data through storytelling, a narrative synthesis was used to interpret patterns and gaps in the literature, providing a comprehensive and contextualized analysis of family-centred early hearing detection and intervention in African settings. The steps taken were as follows:

- Search Strategy

Databases searched included PubMed, Scopus, Web of Science, and Google Scholar. Keywords used included “family-centred care”, “EHDI”, “Africa”, “cultural responsiveness”, and “early hearing intervention”. Sources included were those published in the timeframe 2000 to 2024, with the inclusion of studies focusing on EHDI in African contexts, addressing family-centred approaches, or providing barriers and recommendations specific to Africa.

- Inclusion and Exclusion Criteria

Inclusion criteria included peer-reviewed studies, reviews, and reports related to FC-EHDI, African contexts, and cultural adaptations. Exclusion criteria included studies not related to EHDI or family-centred care, conducted outside Africa, or published in non-English languages. This review focused exclusively on studies examining FC-EHDI within African contexts to ensure that findings and recommendations were directly applicable to the region’s unique sociocultural, economic, and healthcare landscapes. While international programmes offer valuable insights, they often operate within significantly different health infrastructures, policy frameworks, and resource availability. Given the aim of this review—to explore the relevance and responsiveness of FC-EHDI within Africa—limiting the scope to studies conducted in African settings allowed for a more contextually grounded analysis. However, global best practices, such as the FCEI-DHH Principles, were considered where applicable to enhance the discussion and recommendations.

Following the initial search, all retrieved articles were screened for relevance based on their titles and abstracts. Duplicates were identified and removed using reference management software (EndNote 20), ensuring that each study was only included once. A full-text review of the remaining articles was conducted, applying the inclusion and exclusion criteria rigorously to ensure that only studies directly relevant to FC-EHDI in Africa were retained. Studies that did not focus on FC-EHDI, those without sufficient methodological details, and those lacking relevance to the African context were excluded. Where multiple studies reported on similar findings, priority was given to the most recent and comprehensive sources. Any uncertainties regarding inclusion were resolved through discussion among the research team to enhance reliability and minimize bias.

- Data Extraction and Analysis

Data extraction focused on capturing the objectives, methodologies, and key findings and their relevance to FC-EHDI. Thematic synthesis was applied to categorize findings into three primary themes: barriers, evidence supporting FC-EHDI, and recommendations for practice. This process involved: (1) iterative reading of included studies to identify recurring themes; (2) coding of data based on emerging categories such as “resource limitations”, “cultural barriers”, and “community-based interventions”; and (3) synthesizing themes to develop a cohesive understanding of the factors influencing FC-EHDI in Africa.

A thematic analysis approach was used to identify key themes from the included studies. The extracted data were reviewed systematically to identify recurring patterns related to FC-EHDI implementation, barriers, supporting evidence, and recommendations. An inductive coding process was applied, where concepts emerging from the literature were categorized into overarching themes.

To ensure consistency and minimize bias in theme selection, a test-retest reliability process was conducted. The themes were revisited at two different time points to verify the stability of coding decisions. Additionally, external validation was sought by consulting the existing literature and aligning identified themes with established frameworks in FC-EHDI research. This iterative approach strengthened the credibility of the thematic synthesis and ensured that the final themes accurately represented the body of evidence.

- Validation

To ensure the validity and comprehensiveness of the synthesis, the following steps were taken. Firstly, cross-validation of data was conducted, where extracted data were reviewed independently by multiple researchers to ensure consistency and accuracy. Secondly, an iterative synthesis occurred, where themes were revisited and refined through repeated analysis to confirm their relevance and alignment with the study objectives until saturation. Thirdly, peer review was practiced, where the synthesized findings were shared with experts in audiology and public health to verify the interpretations and recommendations. Lastly, discrepancies were addressed, where any differences in interpretation were resolved through consensus discussions among the research team.

## 3. Results and Discussion

### 3.1. Barriers to EHDI in the African Context

EHDI programmes in Africa encounter several barriers that hinder effective implementation (Table 1). These include (1) resource limitations, (2) economic constraints, (3) linguistic and cultural diversity, (4) fragmentation of services, (5) low awareness and advocacy, (6) geographic disparities, (7) stigma and misconceptions, and (8) policy and regulatory gaps [23,24,25,26,27,28,29,30,31]. When it comes to resource limitations, the lack of infrastructure, including audiological equipment and facilities, is a significant documented challenge [5,10,15,23,24,25,26,32,33,34,35]. Many healthcare facilities, particularly in rural areas, are poorly equipped to conduct newborn hearing screenings or provide follow-up care [24]. Additionally, the shortage of trained audiologists and other hearing health professionals further exacerbates the gap in service provision [15]. Similarly, when it comes to economic constraints, the high cost of diagnostic services, hearing aids, cochlear implants, and follow-up care is prohibitive for many families, especially in low-income and rural settings [25,26]. Public healthcare systems often do not cover these costs fully, leaving families to shoulder the financial burden, and very few can afford privately funded healthcare [2,5].

As far as linguistic and cultural diversity are concerned, Africa’s multilingual and multicultural context poses a unique challenge [5,12]. Many EHDI programmes rely on communication in politically dominant languages such as English, Afrikaans, or French, which may not be understood by the majority of caregivers, particularly in rural or underserved communities [27]. This linguistic disconnect undermines the effectiveness of counselling and follow-up care, particularly since there is limited availability of trained interpreters in contexts where practitioners speak languages which are incongruent with the languages spoken by the majority of their patients, as is the case in South Africa [5,16].

When it comes to the fragmentation of services barrier, EHDI services are often not integrated within existing healthcare systems, leading to delays in diagnosis and intervention [28,29]. The lack of coordinated care pathways between maternal and child health services, audiology departments, and early intervention programmes creates inefficiencies and missed opportunities for timely care, as well as limited continuity of care [36]. Additionally, low awareness and advocacy are barriers which arise due to limited awareness of the importance of early hearing detection among both healthcare providers and the general public [23,37,38]. Hearing impairment is often not perceived as an urgent health priority compared to survival-focused conditions, resulting in low levels of advocacy and funding for EHDI programmes [15]. Hearing impairment, taken as low priority for non-survival health issues, is often deprioritized in healthcare agendas, as it is viewed as affecting quality of life rather than survival [39].

Furthermore, when it comes to geographic disparities, rural and remote areas are particularly underserved, with limited access to healthcare services in general and audiology services in particular [39]. Families in these areas face logistical challenges, such as long travel distances and high transportation costs, to access EHDI programmes located in urban centres. As far as stigma and misconceptions go, cultural stigma and misconceptions about hearing impairment and its causes can deter families from seeking help [31]. In some communities, hearing impairment may be attributed to spiritual causes or seen as a source of shame, leading to delays in diagnosis and intervention [30]. Lastly, when it comes to policy and regulatory gaps, the absence of mandated universal newborn hearing screening (UNHS) programmes in many African countries reflects a lack of political commitment and will to address hearing health [40]. Without supportive policies and regulations, the implementation of comprehensive EHDI programmes remains inconsistent and inequitable.

Addressing these barriers requires a multifaceted approach that combines advocacy, capacity building, policy development, and community engagement. By understanding and mitigating these challenges, EHDI programmes can be adapted to better serve the unique needs of African children and their families through FC-EHDI, a responsive framework for Africa.

### 3.2. FC-EHDI: A Responsive Framework for Africa

Family-centred approaches prioritize the active involvement of caregivers in decision-making, service delivery, and follow-up [10]. In African contexts, FC-EHDI aligns with the cultural practice of communal caregiving, where extended family members often play a critical role in child rearing [41]. The key principles of FC-EHDI, as articulated in global consensus statements [42], resonate strongly with African cultural norms:

- Early, Timely, and Equitable Access: Early identification and intervention are crucial. In Africa, community-based screening programmes led by trained healthcare workers can improve access in underserved areas [5].
- Informed Choice and Decision-Making: Empowering families with knowledge in their home language ensures culturally and linguistically appropriate decision-making.
- Family–Provider Partnerships: Reciprocity and mutual respect between providers and families foster trust and engagement, critical in culturally diverse settings.
- Cultural and Linguistic Congruence: FC-EHDI recognizes the importance of providing services in the family’s preferred language and within their cultural context, mitigating communication barriers [22].

FC-EHDI represents a transformative approach tailored to the unique sociocultural dynamics of Africa. At its core, FC-EHDI prioritizes the active engagement of families, recognizing them as equal partners in the intervention process [42]. This framework is particularly well-suited to Africa, where family structures are often extended and caregiving responsibilities are shared among multiple family members, creating a built-in support system. There are several benefits of this approach within the African context: (1) cultural relevance, (2) linguistic accessibility, (3) community-based care, (4) integration of services, (5) empowerment of families, (6) holistic support, and (7) monitoring and feedback.

For cultural relevance, FC-EHDI integrates culturally sensitive practices that respect the values, beliefs, and traditions of families [42]. By involving caregivers in decision-making and recognizing their expertise in understanding their child’s needs, this approach aligns with the communal ethos prevalent in many African societies. Healthcare providers trained in cultural competence can facilitate this alignment, ensuring that interventions are meaningful and acceptable to families. For linguistic accessibility, Africa’s linguistic diversity necessitates the delivery of EHDI services in the home languages of families [5,10]. FC-EHDI promotes the development and use of multilingual resources, enabling caregivers to comprehend and participate fully in their child’s care [24]. Linguistic inclusivity not only enhances communication but also fosters trust and reduces the anxiety often associated with accessing healthcare.

Additionally, the decentralization of EHDI services is a cornerstone of FC-EHDI, thus aligning with community-based care [8]. Task-shifting strategies, where community health workers and other non-specialist providers are trained to conduct basic screenings and provide follow-up care, can significantly expand the reach of EHDI programmes [13,43]. These community-based initiatives leverage existing structures, reduce travel burdens for families, and ensure that services are accessible even in remote areas, and these can be enhanced through the use of digital healthcare, including tele-audiology. Moreover, as far as integration of services is concerned, FC-EHDI emphasizes the seamless integration of hearing care into existing maternal and child health programmes [28,41,44]. This integration ensures that hearing screenings become a routine part of postnatal care, increasing early detection rates. By establishing referral pathways and fostering collaboration among healthcare providers, FC-EHDI minimizes delays and enhances the overall efficiency of intervention programmes.

Furthermore, empowerment of families with knowledge and skills is a central tenet of FC-EHDI [11]. This empowerment includes providing families with information about hearing impairment, available interventions, and the expected outcomes of care [13]. Training caregivers in communication techniques, such as sign language or auditory–verbal strategies, equips them to actively support their child’s development [45]. Additionally, when it comes to holistic support, beyond addressing hearing impairment, FC-EHDI recognizes the need to support families emotionally, socially, and economically [5]. Programmes may include counselling services, peer support groups, and financial assistance to alleviate the multifaceted challenges families face. Holistic support strengthens family resilience and ensures sustained engagement with intervention programmes. Lastly, as far as monitoring and feedback are concerned, regular monitoring of both child and family outcomes is integral to the FC-EHDI framework [42]. Feedback mechanisms enable caregivers to voice their experiences, preferences, and concerns, ensuring that services remain responsive to their needs. This iterative process fosters continuous improvement and reinforces the family-centred philosophy [15].

By adopting FC-EHDI, African healthcare systems can overcome the limitations of traditional models and create equitable, effective, and culturally resonant interventions. This approach not only addresses the technical aspects of hearing care but also embraces the social and cultural dimensions that are critical to achieving meaningful outcomes for children and their families.

### 3.3. Evidence from African Studies

A significant body of research (Table 2), mostly from South Africa, highlights the effectiveness and relevance of FC-EHDI within the African context [2,5,8,24,28,34,46,47,48,49,50,51], highlighting how cultural responsiveness and tailored approaches can improve outcomes for DHH children and their families. This research also underscores the potential of FC-EHDI in addressing the unique challenges faced by families of DHH children in this context.

Kanji [14] documented how incorporating community health workers into hearing screening programmes increased the reach and accessibility of EHDI services in Sub-Saharan Africa. Community-based programmes reduced the financial and logistical burdens families faced and empowered local communities to take an active role in supporting DHH children. In Kenya, Mbogo [50] highlighted the role of extended family systems in supporting DHH children. The study found that involving extended family members in counselling sessions and intervention planning led to more cohesive and sustainable support structures for children. In South Africa, Maluleke, Chiwutsi, and Khoza-Shangase [8] emphasized that caregivers’ active involvement in EHDI programmes enhances the developmental outcomes of DHH children. They found that programmes integrating family-centred principles—such as involving caregivers in decision-making and providing support in the family’s home language—were effective in fostering engagement and satisfaction. Their study highlighted the need for culturally and linguistically congruent services to ensure equitable access and understanding.

Khoza-Shangase and Kanji [46] discussed best practices for EHDI in South Africa, noting the critical role of task-shifting and community-based models in addressing systemic barriers. By training community health workers to conduct screenings and provide counselling, these models expanded access to underserved areas. The study also called for the integration of tele-audiology services to mitigate geographic and resource constraints. Swanepoel and Störbeck [47] advocated for comprehensive advocacy efforts to prioritize hearing health in Africa’s healthcare agenda. They highlighted the importance of family-centred practices in improving the acceptance and sustainability of EHDI programmes, particularly in rural and resource-limited settings.

Maluleke, Khoza-Shangase, and Kanji [5] explored caregivers’ expectations of EHDI services in South Africa, identifying a gap between caregiver needs and the services provided. The study revealed that families value clear communication, timely follow-up, and access to affordable interventions. These findings underline the importance of aligning services with caregivers’ cultural and socioeconomic contexts. Kanji and Casoojee [48] emphasized the value of interdisciplinary collaboration in EHDI programmes. Their research showed that integrating audiologists, speech-language therapists, and educators fosters holistic support for children and families. This approach ensures that developmental, communicative, and educational needs are addressed concurrently.

Ehlert and Coetzer [34] focused on maternal knowledge regarding EHDI, revealing that limited awareness and understanding of hearing impairment hinder timely intervention. This finding highlights the need for targeted educational campaigns to empower caregivers and communities. Khoza-Shangase [51] identified factors compromising EHDI service delivery, including stigma and misconceptions about hearing loss. The study highlighted that addressing these barriers through community engagement and culturally sensitive counselling significantly improves programme acceptance and outcomes.

Moodley and Storbeck [28] provided a comprehensive review of EHDI in South Africa, identifying systemic gaps and opportunities for improvement. They recommended leveraging existing health infrastructure, such as immunization programmes, to integrate hearing screenings and early interventions seamlessly. Lastly, Naidoo and Khan [24] analysed barriers to EHDI in KwaZulu-Natal in South Africa, identifying geographic and economic disparities as significant challenges. They advocated for decentralized service delivery models and mobile health units to reach remote communities effectively.

Overall, research in South Africa mainly highlights the potential of FC-EHDI in addressing gaps in EHDI service delivery. The caregiver-focused studies highlighted above generally reveal three key findings: (1) expectations and experiences, (2) barriers and facilitators, and (3) perceived family-centredness. When it comes to expectations and experiences, delays in diagnosis and intervention often fail to meet caregivers’ expectations; however, services that incorporate caregiver feedback and preferences show higher satisfaction and engagement levels. As far as barriers and facilitators go, while high costs and linguistic barriers hinder access, programmes offering support such as sign language training and informational counselling enhance family participation. Lastly, when it comes to perceived family-centredness, despite challenges, caregivers report greater satisfaction with EHDI programmes that emphasize family-centred principles, particularly in respectful care and enabling partnerships.

Collectively, these studies demonstrate that FC-EHDI approaches, when adapted to Africa’s unique cultural and systemic contexts, can significantly improve outcomes for DHH children and their families. They highlight the importance of community-based care, interdisciplinary collaboration, and culturally responsive practices in ensuring equitable and effective service delivery.

An essential component of FC-EHDI is the opportunity for parents not only to connect with other parents of children who are DHH but also to engage with DHH adults who can provide lived experience perspectives on hearing loss, communication, and identity formation. Parent-to-DHH adult mentorship has been recognized in global FC-EHDI frameworks as a valuable source of guidance, particularly in helping families understand diverse communication options, educational pathways, and social integration experiences. Research outside Africa suggests that these connections help parents navigate their child’s diagnosis with greater confidence while reinforcing positive identity development for the child. However, within the African context, there have been few studies explicitly examining structured parent-to-DHH adult support networks in EHDI programmes. Much of the documented FC-EHDI work in Africa has focused on parent-to-parent peer support and professional-led intervention services, with minimal discussion of the role of DHH adult mentorship in family support systems. This gap may be attributed to various factors, including cultural perceptions of disability, limited visibility of DHH adults in leadership roles, and a historical emphasis on medicalized intervention models over social support frameworks. While anecdotal evidence and advocacy groups suggest emerging efforts to strengthen such connections, further research is needed to explore how integrating DHH adult perspectives into FC-EHDI in Africa could enhance both parental empowerment and the self-advocacy skills of children who are DHH.

### 3.4. Recommendations for Practice

**Figure 1:**
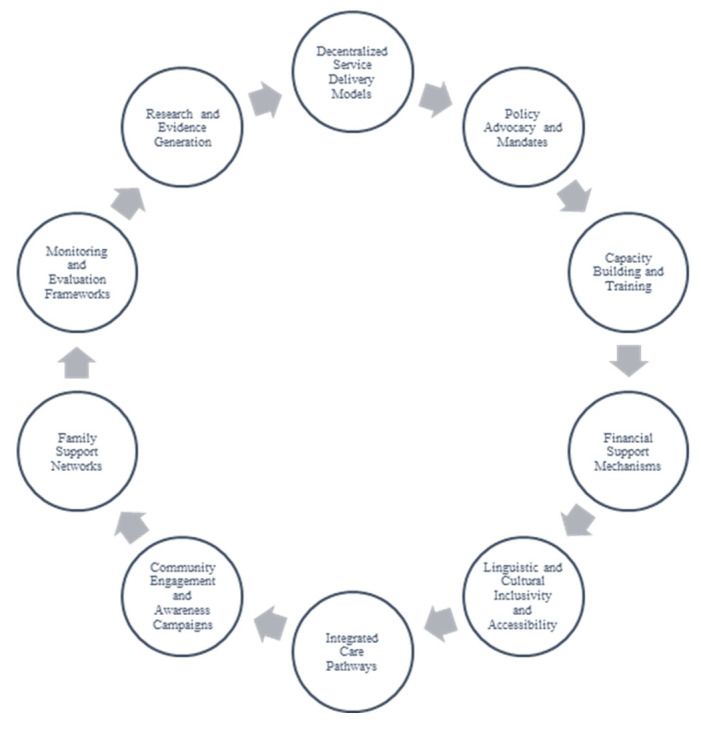
Strategies to enhance implementation of FC-EHDI across Africa.

Paying careful attention to contextual realities within the African context, to enhance the implementation of FC-EHDI across Africa, the following strategies and recommendations, depicted in Figure 1 and Table 3, are proposed in this review.

Decentralized service delivery models in the form of community-based service delivery are important to increase access to hearing care in underserved and rural areas. Training community health workers and other task-shifting cadres to conduct initial hearing screenings and refer families to specialists can address geographic disparities and reduce service delivery gaps. Task-shifting, particularly while leveraging technology, including tele-audiology, can expand the reach of EHDI programmes across Africa. At the same time, policy advocacy needs to be strengthened, with African governments prioritising hearing health in national healthcare agendas and allocating resources to support universal newborn hearing screening (UNHS). Policy advocacy and mandates are crucial, where national policies that mandate UNHS and establish clear referral pathways are formulated and/or implemented [40]. Here, policymakers can prioritize hearing health as a critical component of maternal and child health programmes.

Additionally, capacity building and training is an important strategy to adopt in this context, where governments invest in training healthcare providers, including audiologists, speech-language therapists, Teachers of the Deaf (ToDs), and community health workers (task-shifting cadres) to deliver culturally responsive and family-centred care. Incorporating cultural competence into training curricula ensures services are tailored to the diverse needs of families. Culturally responsive training, where healthcare providers receive training on cultural competence to ensure services align with local norms and values, is important. Linguistic and cultural inclusivity and accessibility are crucial aspects of ensuring appropriate FC-EHDI implementation in this context. Producing multilingual educational materials in local languages and providing interpretation services to address linguistic diversity should form part of the strategy. Ensuring families can access information in their preferred languages enhances understanding and engagement. Furthermore, community engagement and awareness campaigns designed to be culturally appropriate to educate families and communities about the importance of early hearing detection and intervention are crucial. Leveraging local leaders and community-based organizations can increase the reach and impact of these campaigns.

Furthermore, implementation strategies should include financial support mechanisms in which subsidy programmes are developed or hearing health services are included within national health insurance schemes to alleviate the financial burden on families. Donor funding and public–private partnerships can also play a role in bridging funding gaps for hearing aids, cochlear implants, intervention services, and special schooling for DHH children. Similarly, integrated care pathways should be carefully considered. National departments of health within this context should integrate hearing screening and intervention services into existing healthcare frameworks, such as immunization and antenatal care programmes. Seamless integration and referral pathways between screening, diagnosis, and intervention reduces fragmentation, improves efficiency, and reduces delays in care delivery. This also calls for the establishment of family support networks with peer support groups and counselling services for families of DHH children. Sharing experiences and resources within these networks can provide emotional and practical support. Additionally, ToDs play a critical role in ensuring continued support for children with late diagnoses, particularly in contexts where newborn hearing screening is not universally implemented. Given that many children in Africa may only be diagnosed after 12 months of age, ToDs are essential in bridging developmental gaps by providing specialized educational support, language development strategies, and communication access within school settings. Their involvement in early childhood education programmes and school-based interventions will ensure that children who are DHH receive individualized support tailored to their specific learning needs. Strengthening collaboration between ToDs and healthcare professionals can enhance continuity of care, improving long-term educational and social outcomes for children with hearing loss.

Lastly, strategies to enhance the implementation of FC-EHDI in the African context should also include monitoring and evaluation frameworks, where robust monitoring and evaluation systems to track the outcomes of FC-EHDI programmes are implemented. Collecting and analysing data on family satisfaction, intervention efficacy, and service accessibility ensures continuous improvement. This could form part of the research and evidence generation part of the strategy, where research on FC-EHDI to build a stronger evidence base for its implementation in African contexts is encouraged and facilitated. Local studies can inform programme design and highlight successful models for replication.

By addressing these recommendations, African countries can create sustainable, contextually and culturally relevant FC-EHDI programmes that prioritize family involvement and improve outcomes for DHH children and their families.

### 3.5. Future Directions

Looking ahead, the advancement of FC-EHDI in Africa requires an emphasis on innovation, collaboration, and long-term sustainability [52]. This review recommends that future directions should focus on the following key areas:

- Policy and Advocacy Expansion: Advocacy efforts should focus on embedding FC-EHDI within national health policies and securing dedicated funding streams. Policymakers need to be sensitized to the long-term benefits of early intervention, not only for individuals but also for societal productivity and well-being.
- Sustainable Funding Models: Developing partnerships with private sector stakeholders, international organizations, and government agencies will be key to establishing sustainable financing mechanisms.
- Capacity Building: Expanding training programmes to increase the number of skilled professionals in audiology and related fields in the continent is crucial. Incorporating FC-EHDI principles into medical and allied health curricula will ensure a new generation of professionals equipped to deliver family-centred care.
- Scaling Successful Models: Piloted programmes that have demonstrated success, such as task-shifting strategies and community-based screenings, should be scaled up and replicated across different African regions. Adapting these models to the specific needs of various communities will ensure wider reach and impact.
- Integration of Technology: Leveraging technological advancements, such as mobile health (mHealth) platforms, can revolutionize FC-EHDI service delivery within the resource-constrained African context. Mobile apps can be developed to provide remote counselling, facilitate virtual follow-ups, and offer educational resources to families. Tele-audiology services can bridge gaps in access, particularly for families in remote or underserved areas.
- Interdisciplinary Collaboration: Strengthening partnerships between audiologists, speech-language therapists, educators, and community health workers (and other task-shifting cadres) can enhance the holistic nature of FC-EHDI programmes. Collaborative approaches ensure that children receive comprehensive support across different aspects of their development.
- Cultural Adaptation of Interventions: Continuous research into local cultural practices and beliefs will enable the customization of FC-EHDI programmes. Tailoring interventions to respect cultural nuances fosters acceptance and engagement among families.
- Focus on Inclusive Education: As DHH children transition into educational settings, collaboration with schools to promote inclusive education practices will be vital. Training teachers and creating supportive learning environments will ensure that children reach their full potential.
- Empowering Families as Advocates: Building the capacity of families to advocate for their children’s needs will amplify the voice of the community in shaping FC-EHDI policies and practices. Advocacy training can enable families to engage with policymakers and stakeholders effectively.
- Data-Driven Decision Making: Establishing robust data collection systems will enable continuous evaluation of FC-EHDI programmes. Data analytics can identify trends, measure impact, and inform strategies for improvement, ensuring that programmes remain responsive to evolving needs.

These recommended future directions highlight the potential to transform FC-EHDI in Africa, ensuring that it becomes a cornerstone of equitable and culturally relevant healthcare systems. By focusing on innovation, collaboration, evidence-based practice, and sustainability, FC-EHDI can continue to evolve to meet the needs of families and DHH children.

## 4. Conclusions

As advocated by Kanji and Khoza-Shangase [52], FC-EHDI represents a critical step forward in addressing the unique challenges of hearing impairment in the African context. By prioritizing family involvement and cultural responsiveness, family engagement, and innovative practices, FC-EHDI aligns with the values and structures of African societies, offering a sustainable and effective approach to early hearing care. This framework not only addresses technical barriers but also embraces the social and cultural dimensions that are essential for achieving meaningful outcomes, while fostering equity and inclusion. The evidence presented in this review highlights the transformative potential of FC-EHDI when adapted to local contexts. By leveraging community-based care, linguistic inclusivity, and holistic support systems, FC-EHDI enhances accessibility, engagement, and efficacy. The integration of culturally relevant practices ensures that families are empowered and equipped to support their children’s developmental needs.

Looking ahead, the success of FC-EHDI in Africa hinges on sustained advocacy, capacity building, and investment in technology and research. Policymakers, healthcare providers, and community leaders must collaborate to institutionalize FC-EHDI within national health systems and education frameworks. Future efforts must integrate scalable technologies, interdisciplinary collaborations, and sustainable funding models. Innovations such as tele-audiology, mobile health platforms, and inclusive education models can further bridge existing gaps, enabling equitable access to care for all families. Ultimately, FC-EHDI has the potential to reshape the landscape of hearing care in Africa. By placing families at the heart of intervention strategies, this approach ensures that DHH children across Africa can access timely and effective care, supported by their families and communities to achieve their full potential.

## Figures and Tables

**Table 1:** Barriers to EHDI in Africa.

Barrier	Description	Example Studies
Resource Limitations	Lack of equipment, trained professionals, and infrastructure.	Olusanya [23]; Naidoo & Khan [24]
Economic Constraints	High costs of diagnostic tools, hearing aids, and follow-up care.	Casoojee [25]; Rajanbabu et al. [26]
Linguistic Diversity	Challenges due to multilingualism and lack of interpreters.	Tönsing et al. [27]; Maluleke et al. [5]
Fragmented Services	Disjointed care pathways between screening, diagnosis, and intervention.	Moodley & Storbeck [28]; Kanji [29]
Stigma and Misconceptions	Cultural misconceptions about hearing impairment as spiritual or shameful.	Pillay & Moonsamy [30]; Rohwerder [31]

**Table 2:** Evidence Supporting FC-EHDI.

Study	Key Findings
Maluleke et al. [8,9]	Caregivers value culturally and linguistically tailored EHDI services that enhance engagement.
Khoza-Shangase & Kanji [46]	Task-shifting and tele-audiology expand access, addressing geographic barriers effectively.
Swanepoel & Störbeck [47]	Advocacy for policy integration of EHDI into national health systems is crucial for sustainability.
Moodley & Storbeck [28]	Integration with maternal health services, such as immunization programmes, improves outcomes.
Kanji & Casoojee [48]	Interdisciplinary collaboration ensures holistic support for children and families.
Ehlert & Coetzer [34]	Limited maternal knowledge of EHDI highlights the need for targeted educational campaigns.
Naidoo & Khan [24]	Geographic disparities require mobile health units and decentralized care delivery.
Maluleke, Khoza-Shangase, & Kanji [5]	Caregivers expect clear communication, affordable services, and timely follow-ups.
Khoza-Shangase et al. [49]	African perspectives emphasize culturally congruent practices to enhance EHDI efficacy.
Rajanbabu et al. [26]	Systematic review shows that low-resource settings benefit from community-driven EHDI strategies.
Petrocchi-Bartal et al. [35]	Embedding EHDI within broader healthcare systems enhances its reach and effectiveness.

**Table 3:** Recommendations for Practice.

Recommendation	Description	Expected Impact
Decentralized Services	Train community health workers to deliver screenings and counselling.	Increased access in underserved areas.
Linguistic Inclusivity	Develop multilingual educational materials and interpretation services.	Enhanced caregiver understanding and engagement.
Integrated Pathways	Embed EHDI into maternal and child health programmes.	Streamlined and effective service delivery.
Technological Integration	Use tele-audiology and mobile platforms for remote counselling and follow-ups.	Improved efficiency and coverage.
Family Support Networks	Establish peer-led groups and counselling services for emotional and practical support.	Strengthened family resilience and advocacy.

## Data Availability

Data supporting the findings of this study are available within the paper.
